# Functional Connectivity Substrates for tDCS Response in Minimally Conscious State Patients

**DOI:** 10.3389/fncel.2016.00257

**Published:** 2016-11-03

**Authors:** Carlo Cavaliere, Marco Aiello, Carol Di Perri, Enrico Amico, Charlotte Martial, Aurore Thibaut, Steven Laureys, Andrea Soddu

**Affiliations:** ^1^Coma Science Group, GIGA-Research, University and University Hospital of LiegeLiege, Belgium; ^2^NAPLab, IRCCS SDN Istituto di Ricerca Diagnostica e NucleareNaples, Italy; ^3^Spaulding Neuromodulation Center, Spaulding Rehabilitation Hospital/Harvard Medical SchoolBoston, MA, USA; ^4^Department of Physics and Astronomy, Brain and Mind Institute, Western UniversityLondon, ON, Canada

**Keywords:** transcranial direct current stimulation, disorders of consciousness, minimally conscious state, magnetic resonance imaging, resting state networks, prefrontal cortex

## Abstract

Transcranial direct current stimulation (tDCS) is a non-invasive technique recently employed in disorders of consciousness, and determining a transitory recovery of signs of consciousness in almost half of minimally conscious state (MCS) patients. Although the rising evidences about its possible role in the treatment of many neurological and psychiatric conditions exist, no evidences exist about brain functional connectivity substrates underlying tDCS response. We retrospectively evaluated resting state functional Magnetic Resonance Imaging (fMRI) of 16 sub-acute and chronic MCS patients (6 tDCS responders) who successively received a single left dorsolateral prefrontal cortex (DLPFC) tDCS in a double-blind randomized cross-over trial. A seed-based approach for regions of left extrinsic control network (ECN) and default-mode network (DMN) was performed. tDCS responders showed an increased left intra-network connectivity for regions co-activated with left DLPFC, and significantly with left inferior frontal gyrus. Non-responders (NR) MCS patients showed an increased connectivity between left DLPFC and midline cortical structures, including anterior cingulate cortex and precuneus. Our findings suggest that a prior high connectivity with regions belonging to ECN can facilitate transitory recovery of consciousness in a subgroup of MCS patients that underwent tDCS treatment. Therefore, resting state-fMRI could be very valuable in detecting the neuronal conditions necessary for tDCS to improve behavior in MCS.

## Introduction

Transcranial direct current stimulation (tDCS) is a non-invasive technique for the stimulation of the cerebral cortex that modulates the spontaneous firing rate of neurons through a weak, constant and direct current applied to the scalp surface (Nitsche and Paulus, 2000).

Although tDCS mechanisms are only partially understood (Stagg and Nitsche, 2011), numerous studies have evaluated the effects of tDCS in the treatment of several neurological and psychiatric diseases, including depression (Kalu et al., 2012), tinnitus (Langguth and De Ridder, 2013) and Parkinson’s disease (Boggio et al., 2006). In particular, anodal stimulation of the left dorsolateral prefrontal cortex (DLPFC) was shown to improve performance of several cognitive abilities in healthy subjects (Iyer et al., 2005; Fiori et al., 2011) and in patients with stroke (Kang et al., 2009) or Alzheimer’s disease (Ferrucci et al., 2008). Conversely, a very few studies have employed this technique in patients suffering from disorders of consciousness (Angelakis et al., 2014; Thibaut et al., 2014, 2015; Naro et al., 2015).

Although this complex syndrome has a heavy impact on the health system, patient’s bedside clinical assessment is rather tricky (Majerus et al., 2005; Schnakers et al., 2009) and multimodal neuroimaging integration is often required for a correct diagnosis and prognosis evaluation of these patients (Di Perri et al., 2014; Giacino et al., 2014). Moreover, although the efforts spent to identify patients that could emerge from this state and recover consciousness (e.g., EMCS; Bruno et al., 2012), no evidence-based guidelines for the treatment of this condition has been standardized (Bernat, 2006).

In a recent trial with a single tDCS on the left DLPFC, our group reported an improvement of consciousness level in 13 out of 30 studied patients (43%) in a minimally conscious state (MCS; Thibaut et al., 2014). In a following study (Thibaut et al., 2015), same authors characterized patient responders to tDCS of left DLPFC for their relative gray matter preservation on VBM analysis and residual brain metabolic activity on FDG-PET examination.

Despite these first positive reports about tDCS application in MCS, no studies have investigated putative resting state network (RSN) changes that could explain different response to tDCS in these patients. In healthy subjects, DLPFC is a region recruited in multimodal extrinsic control network (ECN) that includes lateral frontal and parietal cortices and it is involved in external awareness (Greicius et al., 2003). ECN is physiologically anti-correlated to, and in competition with, another RSN called default-mode network (DMN), encompassing the posterior cingulate cortex/precuneus, the medial prefrontal cortex, and bilateral temporoparietal junctions, and involved in self-awareness (Greicius et al., 2003; Tian et al., 2007). Both networks have been demonstrated to be differently altered in disorders of consciousness (Boly et al., 2008; Vanhaudenhuyse et al., 2011; Guldenmund et al., 2012; Heine et al., 2012; Crone et al., 2013), and partially restored with the recovery of consciousness (Laureys and Schiff, 2012). For this reason it appears crucial to investigate the role played by functional connectivity during resting state, and in particular left ECN, in MCS patients responding to DLPFC and tDCS with a transient recovery of signs of consciousness.

The aim of this study is to investigate resting state functional connectivity of left DLPFC in MCS patients that underwent a single session of anodal tDCS, in order to retrospectively evaluate functional connectivity patterns predictive for stimulation response and, more importantly, temporary recovery of some signs of consciousness.

## Materials and Methods

### Patients

Based on the previous study (Thibaut et al., 2014), traumatic and non-traumatic patients in a sub-acute and chronic MCS (>28 days), as diagnosed following previously published criteria (Giacino et al., 2002), were included. Patients with a metallic cerebral implant or pacemaker (in line with the safety criteria for tDCS (Nitsche et al., 2003) or contra-indications to MRI examination) and patients who received sedatives and other drugs that could alter functional Magnetic Resonance Imaging (fMRI) signal (Liu et al., 2015) and/or response to tDCS (Stagg and Nitsche, 2011) were excluded.

Out of the 30 MCS patients included in our previous study (Thibaut et al., 2014), 19 patients underwent a brain resting-state fMRI acquisition, as part of their diagnostic and prognostic workout in our tertiary expert unit (Stender et al., 2014). The fMRI scans of three patients (1 responder and 2 non-responders (NR)) were excluded from the statistical analysis due to suboptimal normalization (see below).

MR scans were performed in resting-state conditions within 1 week prior to tDCS. The study was approved by the ethics committee of the University and University Hospital of Liege, Belgium (ClinicalTrials.gov NCT01673126), and written informed consent was obtained by the legal representative.

Active and sham tDCS were applied for 20 min and tested in randomized order in two separate sessions separated by 48 h, as previously published (Thibaut et al., 2014, 2015). Direct current was applied using surface electrodes with the anode (i.e., active electrode) placed over the left DLPFC (F3 according to the 10-20 international system (Herwig et al., 2003)) and the cathode (i.e., reference electrode) positioned over the right supraorbital region. During active tDCS, the current was increased to 2 mA, the maximum allowed according to the safety guidelines (Nitsche et al., 2003). For the sham condition, the same electrode placement was used as in the stimulation condition, but the current was applied for 5 s at the beginning and the end of the stimulation and was then ramped down. Impedances were kept <10 kΩ and voltage <26 V.

**Figure 1:**
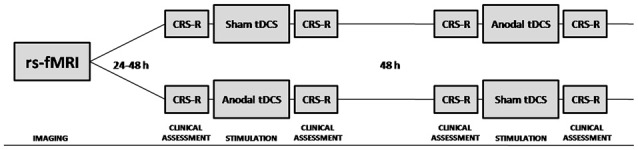
**Schematic representation of the study protocol.** Resting state functional magnetic resonance imaging (rs-fMRI) was performed 24–48 h prior to inclusion in the double-blind randomized cross-over transcranial direct current stimulation (tDCS) trial. Behavioral signs of consciousness were assessed by means of standardized Coma Recovery Scale Revised (CRS-R) assessments, performed before and directly after the anodal tDCS and sham tDCS sessions.

tDCS responders were defined by the recovery of at least one additional sign of consciousness after tDCS, that was never present before real tDCS, nor before or after the sham tDCS session (Thibaut et al., 2014, 2015). Behavioral signs of consciousness were assessed by means of standardized Coma Recovery Scale Revised (CRS-R) assessments (Giacino, 2004), performed before and directly after the anodal tDCS and sham tDCS sessions (Figure 1). The CRS-R consists of 23 hierarchically arranged items that comprise six subscales addressing auditory, visual, motor, verbal, communication and arousal functions. The lowest item on each subscale represents reflexive activity, whereas the highest items represent cognitively mediated behaviors.

### MRI Data Acquisition

In all subjects, resting state fMRI data were acquired on a 3T magnetic resonance scanner (Trio Tim Siemens, Erlangen, Germany). Resting state functional magnetic resonance imaging (rs-fMRI) images were obtained with a gradient echoplanar sequence using axial slice orientation with 32 slices, field of view (FOV) 192 mm^2^ × 192 mm^2^, voxel size 3 mm^3^ × 3 mm^3^ × 3 mm^3^, matrix size 64 × 64 × 32, repetition time (TR) = 2000 ms, echo time (TE) = 30 ms, flip angle (FA) = 78°. Head movements were minimized using customized cushions.

Structural MRI T1 data were acquired performing a T1-weighted 3D gradient echo images sequence using 120 slices, TR ~2300 ms, TE ~2.47 ms, voxel size ~1 mm^3^ × 1 mm^3^ × 1.2 mm^3^, FA ~9, FOV ~256 mm^2^ × 256 mm^2^.

In addition, during the same scanning session, axial T2-FLAIR, T2-TSE and diffusion weighted images were also acquired for a comprehensive assessment of anatomical alterations.

### MRI Data Processing

MRI data were pre-processed using the DPABI 4.0 (Chao-Gan and Yu-Feng, 2010; Aiello et al., 2015), a Matlab (Mathworks Inc.) toolbox containing libraries for fMRI analysis that relies on the Statistical Parametric Mapping 8 package (SPM8, the Wellcome Department of Neurology, London UK (Friston and Frith, 1995).

The first 10 time points of rs-fMRI images were removed to avoid non-equilibrium effects of magnetization. The remaining 230 volumes of functional BOLD images were corrected for slice timing effects and motion correction was performed by aligning all the subsequent volumes to the first time point (Friston and Frith, 1995). Studies with an estimated maximum head motion larger than 3.0 mm and/or 3.0° were excluded.

In order to remove BOLD signal fluctuations unrelated to neuronal activity, the white matter and cerebrospinal fluid mean signals were preliminarily regressed out as nuisance variables (Zuo et al., 2013). In addition, to take into account signal drifts that arise from scanner instability or other possible causes, linear trend was analogously removed from each voxel’s time course. Each volume was finally spatially normalized to the Montreal Neurological Institute (MNI) template at voxel size of 3 mm^3^ × 3 mm^3^ × 3 mm^3^: after a co-registration between rs-fMRI and T1 images of each subject, the spatial transformation from single subject to MNI space at voxel size of 3 mm^3^ × 3 mm^3^ × 3 mm^3^ was derived from T1-weighted high resolution data by means of the diffeomorphic normalization step performed during the DARTEL segmentation procedure (Ashburner, 2007) implemented in SPM 8.

RSNs were extracted from pre-processed rs-fMRI data by means of seed-based analysis performed within DPARSF toolbox. The seeds for the left ECN were defined as spheres with radius of 6 mm and centers placed over left DLPFC at (−32, −11, 60) for DL1 and (−44, 7, 22) for DL3 as suggested in Taren et al. (2011). The seed for DMN was defined as a sphere with radius of 6 mm with MNI centroid coordinates (0, −50, 28) mm, as derived from an independent dataset of healthy participants (Jovicich et al., 2016). Voxel-wise maps relative to each RSN were generated considering the Pearson correlation coefficient between the time course of each voxel and the time course averaged over the seed sphere. RSN maps were finally smoothed with an isotropic gaussian filter of 8 mm (FWHM) in order to compensate for normal variation across subjects.

### Statistical Analysis

Differences between the imaging variables of responders and NR groups were assessed by means of two sample *t*-test as implemented in SPM. Both responders (R) > NR and responders (R) < NR contrasts were assessed. The results were considered statistically significant under *p* < 0.05 family wise error (FWE) corrected at cluster level, with clusters made of voxels surviving a *p* < 0.01 with minimum cluster extent of 50 voxels.

## Results

### Clinical

Out of the 16 patients in sub-acute or chronic MCS that were included in the analyses, six were tDCS responder (3 post-traumatic, 3 non-traumatic; 3 men) and 10 were NR (7 post- traumatic, 3 non-traumatic; 6 men). The responders and NR did not show a significant difference in age (mean ± SD; 42 ± 17 vs. 35 ± 13 years respectively; *p* = 0.38), time since onset (7 ± 9 vs. 3 ± 3 years; *p* = 0.22), or baseline CRS-R total score (median (IQR); 9(3) vs. 7(4); *p* = 0.7). After active tDCS of left DLPFC, MCS patients in the responders group transiently improve signs of consciousness, as assessed by CRS-R total scores, showing an increase from 2 to 4 points after the anodal tDCS session, and never present before real tDCS, nor before or after the sham tDCS session. No effect of tDCS on any of the CRS-R subscales was observed in any group. No tDCS-related side effects were observed.

### Resting-State fMRI

When looking at the site of stimulation, the left DLPFC, we decided to use two different seeds, according to the topographic organization of different functions (Taren et al., 2011).

DL1 seed showed no significant changes in the related functional connectivity patterns when we compared responders to NR group.

**Figure 2:**
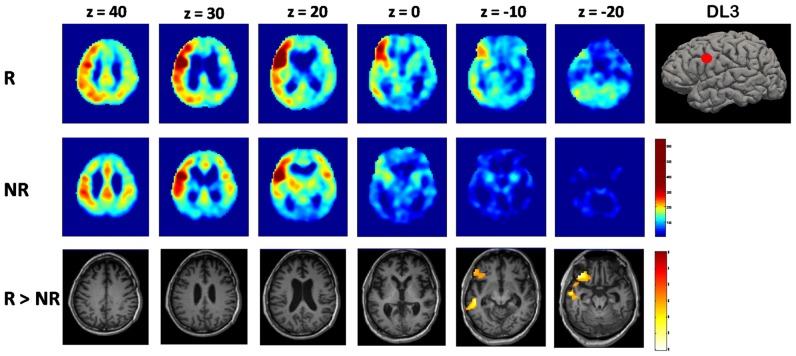
**Voxel-wise maps of functional connectivity of the left dorsolateral prefrontal cortex (DLPFC; DL3) seed.** A brain volume rendering with the cortical DL3 seed position is represented on the right. Each map is resulting from averaging across subjects of each group (responders (R) and non-responders (NR)). Functional connectivity intensity at each voxel is represented as the pearson’s correlation coefficient between the rs-fMRI signal of the DL3 spherical ROI and the voxels of the rest of the brain. On the bottom line, brain areas showing higher coactivation with DL3 seed in six tDCS responders vs. 10 tDCS NR (R > NR; results are family wise error (FWE) corrected for multiple comparisons).

Using DL3 seed, instead, a different functional connectivity pattern was evident for the two groups. In the responders group, voxel-wise average maps were close to physiological left ECN, showing a diffuse asymmetrical co-activation of left lateral fronto-parietal cortices. Differently, NR group showed a reduced left ECN connectivity with a more diffuse and bilateral co-activation of cortical structures, including anterior cingulate cortex and precuneus (Figure 2).

When comparing statistically the two groups, responders showed significantly higher (*p* = 0.007, FWE corrected at cluster level) connectivity between DL3 and left inferior frontal gyrus with respect to the NR group (Figure 2).

When looking at functional connectivity maps originated by precuneus seed and DMN, no significant differences (FWE-corrected at cluster level) were detected between the two groups.

## Discussion

In this study, we retrospectively evaluated functional connectivity patterns of several sub-acute and chronic MCS patients, before the treatment with a single session of tDCS in the left DLPFC. In particular, we investigated with a seed-based approach functional connectivity analysis differences that could provide new insights about the response mechanism to cortical stimulation and the transitory recovery of consciousness.

The DLPFC is a region connected to different cortical and subcortical brain areas, including the orbitofrontal cortex, the thalamus and the parietal and the frontal associative cortices. This area is involved in the functional ECN, known to be related to external awareness (D’Esposito et al., 1998; Lieberman, 2007).

Numerous studies have suggested that the brain’s baseline activity that modulates awareness is related to a widespread set of fronto-parietal associative areas, both on the convexity (“extrinsic system” including DLPFC) and on the midline (“intrinsic system”; Tian et al., 2007; Boly et al., 2008). These two networks are usually negatively correlated in healthy subjects and it has been demonstrated a reciprocal competitive role for these two systems in activation studies (Boly et al., 2007).

In order to investigate functional connectivity differences related to DLPFC stimulation, we decided to use a seed-based approach in our patients. Recently, a topographic pattern for DLPFC connectivity has been proposed, resulting in a subdivision of this cortical region in four functional domains (Taren et al., 2011). Among this, DL1 and DL3 seeds seemed more appealing for our hypothesis, considering the relation between these subregions and stimulus or context effect, respectively (Koechlin et al., 2003). In MCS patients responding to tDCS, compared to NR, functional connectivity analysis using DL3 seed showed an increased connectivity of DLPFC with the inferior frontal gyrus, another cortical region belonging to the left ECN. This increased basal intra-network connectivity detected only for the patients that will benefit from tDCS, responding with a transitory recovery of consciousness, is in line with previous work demonstrating: (i) a disrupted fronto-parietal network in patients with disorders of consciousness (Boly et al., 2008; Vanhaudenhuyse et al., 2011; Guldenmund et al., 2012; Heine et al., 2012; Crone et al., 2013); (ii) a metabolic and gray matter preservation of these regions in tDCS responders (as detected by FDG-PET and voxel-based morphometry-MRI; Thibaut et al., 2015); and (iii) a restoration of intra-network connectivity mediated by thalamus that parallels with recovery of consciousness (Laureys and Schiff, 2012). Moreover, this finding is supported by a recent division of MCS patients into two behavioral and prognostic groups (MCS− and MCS+) that showed more preserved cerebral metabolism in left-sided fronto-parietal cortices for patients with higher probability to recover consciousness (Bruno et al., 2012).

When we look to the average maps for DL3 functional connectivity in both the groups, other qualitative discrepancy became evident. While responders group showed a physiological asymmetrical pattern with an increased co-activation of left fronto-parietal cortices, NR showed a more diffuse and bilateral connectivity pattern with an increased positive co-activation of median structures, like precuneus, belonging to the physiological anti-correlated intrinsic system. In this context, numerous studies have demonstrated not only the competing character of the two systems (Boly et al., 2007; Tian et al., 2007; Vanhaudenhuyse et al., 2011), but also that high prestimulus baseline activity in the intrinsic system is associated with a tendency to ignore environmental stimuli (Sapir et al., 2005). The higher co-activation of midline structures in the NR group could sustain a stronger competitive effect of precuneus over external stimulations, when compared to the other group.

Finally, several authors have analyzed the effects of prefrontal tDCS on resting-state fMRI patterns in 13 healthy subjects (Keeser et al., 2011). Looking for the effects of tDCS on the left ECN, these authors reported an increased co-activation between regions within the frontal lobe and the parietal lobe, cortical regions receiving DLPFC projections (Hagmann et al., 2008; Greicius et al., 2009). Moreover, this and other studies (Nitsche and Paulus, 2000; Lang et al., 2005; Nitsche et al., 2008) confirm that the cerebral effects of a single tDCS session are expected to be stable for about 50 min. Therefore, a further fMRI session following challenging response evaluation could be un-informative in MCS patients, considering that CSR-R assessment is not immediate and it lasts about 90 min. Moreover, movement artifacts deeply affect test-retest of fMRI data of these patients, especially because anesthesia cannot be performed due to its effects on functional connectivity (Bonhomme et al., 2016). In our study, we reported that functional connectivity pattern highlighted by Keeser et al. (2011), when pre-existing to tDCS of left DLPFC, could be predictive of response in MCS patients. This speculation is strengthened by the physiological tDCS effects that modulate the spontaneous firing rate of preexisting brain neuronal networks without inducing the firing of otherwise resting neurons (Stagg and Nitsche, 2011).

Several limitations affect this analysis. The limited size of the population and the high degree of variability within the groups (e.g., neuroradiological findings and etiology), exclude from one side the possibility to generalize our conclusions to other patients, limiting the significance power of this study (Woo et al., 2014), and on the other side limit the chance to predict tDCS response at the individual level. Furthermore, it was not possible to determine exactly the stimulation area or seeds’ position considered the altered anatomy of these patients due to brain lesions, atrophy, cerebral edema and/or scars that might have occurred and deformed the brain. This consideration could for example explain/bias the lack of significant findings determined for DL1 seed. Nevertheless our study showed that rs-fMRI could be valuable in detecting the neuronal conditions necessary for tDCS to improve behavior in MCS patients.

## Author Contributions

CC and MA have analyzed and interpreted results, and drafted the manuscript. CDP and AT have designed the study, followed data acquisitions and critically revised the manuscript. EA and CM have followed data acquisitions and revised the manuscript. SL and AS have critically revised the manuscript and approved the final version to be published.

## Conflict of Interest Statement

The authors declare that the research was conducted in the absence of any commercial or financial relationships that could be construed as a potential conflict of interest.
